# Synthesis of Chitosan/Carbon Nanotubes Composite Films as Potential Removal of Anionic and Cationic Dyes in Aqueous Solutions

**DOI:** 10.1021/acsomega.5c03164

**Published:** 2025-07-17

**Authors:** Emanuele Ferreira Lessa, Rafael Gerhardt, Viviane Arabidian, Keli Arruda da Silva, Nauro da Silveira Junior, Débora Pez Jaeschke, Tito Roberto Sant’Anna Cadaval Junior, Luiz Antonio de Almeida Pinto

**Affiliations:** Industrial Technology Laboratory, School of Chemistry and Food, Federal University of Rio Grande – FURG, Italia Avenue, km 08, Carreiros District, 96203-900 Rio Grande, RS, Brazil

## Abstract

In response to growing environmental concerns about dye-contaminated wastewater, this study introduces a novel approach to enhance the performance of chitosan-based adsorbents by incorporating carbon nanotubes into film matrices. The chitosan/carbon nanotubes (CN/CNTs) films were prepared via the casting technique using the same amount of chitosan but with varying proportions of CNTs (0.1, 0.3, and 0.5% w w^–1^). The resulting films showed uniform thickness, progressive darkening with increasing CNT content, and improved mechanical strength (up to 83.3 MPa) and elongation capacity (up to 29.3%) with higher CNTs concentration. Adsorption studies highlighted the influence of pH on dye removal, with maximum adsorption capacities of 800 mg g^–1^ for crystal violet (pH 8, CS/CNT 0.5%) and 660 mg g^–1^ for tartrazine yellow (pH 2, CS/CNT 0.1%). The PSO model provided the best fit for the kinetic data in all conditions studied, with *k*_2_ varying from 0.0003 to 0.00053 g mg^–1^ min^–1^. For crystal violet, the Henry model best fit the experimental data with *k*_H_ values ranging from 4.34 to 5.85 L g^–1^. In contrast, the Freundlich model was the most appropriate for yellow tartrazine, showing *k*_F_ values between 8.11 and 20.23 (mg g^–1^)­(mg L^–1^)^−1/*n*^. Desorption tests using NaOH solutions demonstrated reusability, with a performance loss of 12% for crystal violet and 6% for yellow tartrazine after four cycles. These findings underscore the potential of CS/CNTs films as sustainable, high-capacity adsorbents for wastewater treatment applications, advancing the design of biopolymer-based composite materials for environmental remediation.

## Graphical abstract

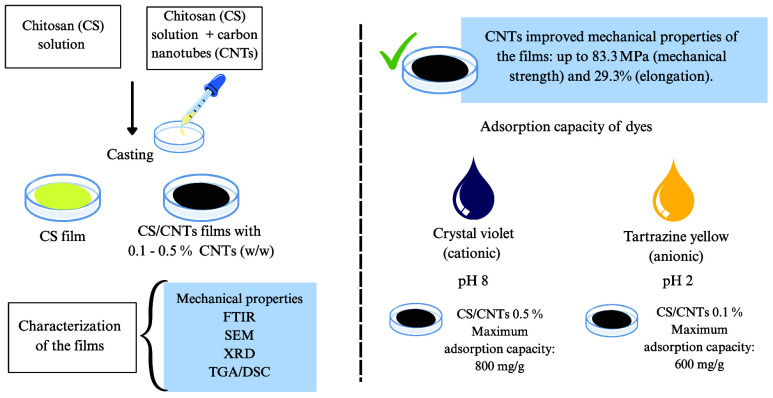

## 1 Introduction

Meeting the growing demands for energy, food security, human health, and biodiversity conservation has become an ongoing global challenge, intensified by rapid population growth and unsustainable industrial practice. Among the most pressing environmental concerns is the contamination of aquatic environments due to improper destinations and inadequate disposal of large proportions of effluents from industrial activities in various sectors, such as pharmaceuticals, agriculture, and textiles.^1–5^ Studies have shown the presence of contaminants, including synthetic dyes and drugs, at low concentrations (ng/L or mg/L) in aquatic environments. Synthetic dyes discarded by the textile and food industries are especially problematic, as they change the natural color in water, reducing sunlight penetration and lowering dissolved oxygen levels.^6^ Particularly, crystal violet and yellow tartrazine dyes pose serious health risks. Crystal violet may cause respiratory issues, skin and eye irritation, and gastrointestinal symptoms. Even at concentrations as low as 1 ppb, it can be toxic and potentially mutagenic to humans and animals.^7^ Moreover, toxicological studies have linked yellow tartrazine exposure to allergic reactions such as asthma, hives, and dermatitis, as well as potential hyperactivity and neurobehavioral changes. Prolonged intake, even at low doses, can damage vital organs like the liver, kidneys, and intestines.^8^ Therefore, effective removal strategies are essential to reducing its harmful impacts.

Many treatment methods are conventionally used to purify water but do not allow for the reuse of effluents, resulting in concerns about the scarcity of this essential resource. Advanced treatment alternatives must be considered to improve the effluent quality that will return to the environment. Bonilla–Petriciolet et al.^9^ reported that several methods for eliminating and treating effluents have been investigated. However, there are still difficulties in treating water to avoid the formation of new contaminants. In the literature, it has been demonstrated that adsorption is promising and low-cost, especially when using eco-friendly and renewable adsorbents that can bind to contaminants through physicochemical mechanisms.^10–12^ Although adsorption is a well-established technique, numerous studies continue to explore novel sources for synthesizing adsorbent materials. Quesada et al.^13^ reported that research on adjusting adsorption parameters for low-cost processes and the effective removal of specific contaminants remains an attractive topic.

In this context, chitin is a renewable biopolymer found in shrimp shells and crustacean exoskeletons. This material is commonly used to produce chitosan, which is a biodegradable and environmentally friendly material. The chitosan molecular structure contains hydroxyl, carboxyl, and amino functional groups, contributing to its diverse applications. Rinaudo^14^ and Crini and Badot^15^ reported that chitosan is considered a promising material for preparing adsorbent composites due to the ease of forming films and modifying its structure.

However, these materials have some characteristics that need to be improved, such as mechanical properties and rapid degradation under humid conditions, which restrict their applications. Another limitation of polysaccharide-based materials, such as chitosan, is their tendency to swell, which can alter their structure and reduce effectiveness. These materials also suffer from low stability, poor reusability, and limited solubility, often requiring organic acids, such as acetic acid, for dissolution. The properties of chitosan-based adsorbents are influenced by factors such as molecular weight, degree of N-acetylation, and the pH of the surrounding solution. While chitosan is effective in removing heavy metals, its performance is limited affinity for the extraction of basic dyes.^16^

An alternative to relying solely on biopolymers for adsorbent preparation is the integration of nanomaterials into polymer matrices. Materials such as chitosan can interact with nanomaterials, enhancing their inherent properties. Studies have demonstrated that nanomaterials such as carbon nanotubes (CNTs) improve the structural characteristics of polymers and act as active adsorption sites, offering increased efficiency and flexibility for structural modifications.^17–19^

CNTs are advanced adsorbents capable of effectively capturing trace contaminants in aquatic environments. Their application in adsorption studies has expanded due to their nanometric scale, uniform porosity, high specific surface area, and mechanical resistance.^20–22^ However, the pure CNTs powder used for adsorption is neither economical nor technically viable, as separating the powder is difficult, and centrifugation is required to remove it completely.^23^ Moreover, CNTs are hydrophobic and agglomerate in aqueous solution. Therefore, to improve their affinity, CNTs are commonly functionalized with different materials, such as chitosan, that improve their dispersion.^24,25^ Ghobashy et al.^26^ developed carbon nanotube aerogels from cross-linked polyacrylamide/chitosan hydrogels to remove pump oil and organic solvents. Characterization showed that CNTs incorporation significantly improved the surface area (up to 316 m^2^/g) and compressive strength (up to 8.677 MPa) while also enhancing hydrophobicity. Moreover, Wei et al.^27^ demonstrated that the incorporation of 1 % (w/w) multiwalled CNTs into chitosan hydrogel significantly enhanced the adsorption of Cd (II) compared to the hydrogel without nanotubes.

Therefore, this study aimed to produce chitosan-based adsorbents with carbon nanotubes addition. The films were prepared through the casting technique with the same amount of chitosan with the proportions of CNTs. The chitosan films with carbon nanotubes were characterized through Fourier transform infrared spectroscopy (FTIR), scanning electron microscopy (SEM), X-ray diffraction (XRD), thermogravimetric analysis (TGA), differential scanning calorimetry (DSC), and point of zero charge (PZC), and their characteristics as adsorbents, such as thickness, color, and mechanical properties, were investigated. Yellow tartrazine food dye and crystal violet textile dye were used as experimental contaminant models for the adsorption tests to evaluate the pH effect. Moreover, to better understand the adsorption of both dyes, kinetics, thermodynamics, and the reuse of films were also studied. These experiments were conducted using the best film composition and pH conditions identified for each dye.

## 2 Materials and Methods

### 2.1 Materials

Shrimp waste was sourced from commercial fish markets in the port region of Rio Grande (RS, Brazil). Multiwalled carbon nanotubes functionalized with (−OH) groups were purchased from Nanostructured & Amorphous Materials, Inc. (USA), with purities higher than 90%, an external diameter ranging from 50 to 80 nm, and an internal diameter ranging from 5 to 15 nm. All reagents used in the analyses were of analytical grade.

### 2.2 Obtaining and Characterization of Chitin and Chitosan

Shrimp waste (*Penaeus brasiliensis*) was used as raw material, and chitin was extracted through chemical treatment following the methodology described by Moura et al.^28^ This methodology includes demineralization (HCl, 2.5 g L^–1^), deproteinization (NaOH, 5.0 g L^–1^), and deodorization (NaClO, 0.4 g L^–1^) steps, followed by drying in a tray dryer to reach a moisture content of 5.0–6.0% (wet basis). Chitosan was obtained through the deacetylation of extracted chitin followed by drying.^28^ The chitin was treated with a NaOH solution (421 g L^–1^) at 130 °C for 2 h under constant stirring to remove acetyl groups and convert it into chitosan. After deacetylation, the material was repeatedly washed with distilled water until neutral pH. Afterward, chitosan was purified into a paste form and dried using a spouted bed. For that, hot air was introduced through the base of a drying column to suspend and fluidize the paste particles, ensuring efficient heat transfer and uniform drying.

### 2.3 Characterization of Chitosan

The ability of the produced chitosan to act as a starting material in the development of composite films was assessed by determining the degree of deacetylation, molar mass, and moisture content. The degree of deacetylation was determined to classify the polymer as either chitin or chitosan. In this study, the linear potentiometric titration method was used.^28^ The conversion of chitin into chitosan was confirmed when the degree of deacetylation determined for chitin was higher than 70%, which is when the material becomes soluble in acidic solution.^14^ The degree of deacetylation is crucial in determining the characteristics of chitosan, as it identifies the polysaccharide structural profile by analyzing the content of N-deacetylated groups and amino groups within the polymer chain. The molar mass of the polymer was determined using a capillary viscosimeter, and the samples used to determine the moisture content were analyzed in triplicate.^29^

### 2.4 Synthesis of the Adsorbent Films

Films were prepared with a 4% (w v^–1^) chitosan solution, using chitosan with an approximately 84% degree of deacetylation and a molar mass of 162 kDa as the polymeric matrix. The films were made using the casting technique, according to the procedure of Moura et al.^28^ and Dotto et al.^29^ Chitosan single (CS) film was prepared as a control by dissolving 2 g (dry basis) of chitosan powder in 50 mL of 1% (v v^–1^) acetic acid solution with stirring at 400 rpm and a magnetic stirrer at room temperature (25 °C) for 4 h. Afterward, 50 mL of the film-forming solution was poured into a Petri dish with a diameter of 14.7 ± 0.2 cm. The films were obtained by evaporating the solvent in an oven with forced air at 40 °C for 24 h; later, they were removed from the plates and stored in desiccators at 25 ± 1 °C for 48 h.

The methodology for preparing chitosan/carbon nanotubes (CS/CNTs) hybrid films followed the same procedure as the chitosan single film (CS), with the addition of varying concentrations of carbon nanotubes to the film-forming solution: 0.1, 0.3, and 0.5% (w w^–1^). After preparation, the resulting films were properly characterized and identified as CS, CS/CNT 0.1%, CS/CNT 0.3%, and CS/CNT 0.5%.

### 2.5 Characterization of Chitosan Single (CS) Films and CS/CNTs Composite Films

The CS films and CS/CNTs composite films were characterized to investigate their thickness, mechanical properties, optical properties, (color), thermal properties, morphology (crystallinity and chemical structure), and surface area (zero charge point), all of which are relevant for their potential application in the adsorption of textile and food dyes.

Thickness is a relevant characteristic for analyzing the film mechanical resistance. The film thickness was determined by using a digital micrometer (Mitutoyo Corp., MDC-25S, Japan) with a resolution of 0.0010 mm. According to Ferreira et al.,^30^ the thickness of each obtained film was calculated as the average of six measurements taken from different regions of the samples.

The mechanical properties of the tensile strength and elongation were measured. The films were characterized by the ASTM method,^31^ using a texturometer (Stable Micro Systems, TA-XT-2i, U.K.). The film samples were cut to 25 × 100 mm^2^ (width × length) and fixed to the equipment with a load of 50 N at approximately 50 mm and a pulling speed of 2 mm s^–1^. This analysis determines the maximum load and length the film can reach before breaking.

The color parameters of the films were carried out based on Munsell’s studies, and the International Commission on Illumination (CIE) developed methods, standards, and principles to determine colors. The CIELAB color space, or the L* a* b* color system, has been used to identify and quantify color attributes or deviations from a standard color. The hue angle was evaluated by eq 1 (0° = red; 90° = yellow; 180° = green; 270° = blue), according to Rhim et al.^32^

1 $$H_{\mathrm{ab}}=\mathrm{ta}n^{-1}\left(\frac{b^{*}}{a^{*}}\right)$$

where *H*_ab_ is the hue angle and a* and b* are the color parameters of the samples.

X-ray diffraction (XRD) analysis was carried out according to Guerra et al.^33^ to determine the crystallinity patterns of the film samples. A diffractometer (Bruker, D-8, Germany) with Cu Kα radiation was used. The operating conditions were 40 kV and 40 mA, and the diffractograms were acquired in the 2θ range from 5 to 120° at a 2 min^–1^ resolution of 0.02°.

The thermal characteristics of the films were determined via TGA and DSC analyses.^34^ Thermogravimetric (TGA) analysis was performed by using a thermobalance (Shimadzu, TGA-60, Japan) with a nitrogen flow of 50 mL min^–1^ at a heating rate of 10 °C min^–1^. The samples were placed in aluminum crucibles and heated to 35–550 °C. Using TGA, it is possible to evaluate the stability and thermal properties of films, allowing the measurement of the variation in mass of a sample as a function of temperature and/or time. When these materials are subjected to heat treatment, their structures may change, characterized by the scission of chemical bonds in the chains. Differential scanning calorimetry (DSC) analysis defines the thermal characteristics of materials. For calorimetry analysis, the samples were previously weighed in hermetically sealed aluminum crucibles and subjected to heating in the temperature range between 20 and 200 °C at a heating rate of 10 °C min^–1^. Using a calorimeter (Shimadzu, DSC-60, Japan) in a nitrogen atmosphere with a gas flow rate of 50 mL min^–1^, curves were obtained that describe the thermal stability profile of the films, presenting data on the material decomposition temperature and peak characteristics of the enthalpy variation.

Fourier transform infrared (FTIR) analysis was conducted according to Daneshvar et al.,^35^ using a Shimadzu spectrometer (Model Affinity, Japan) operating in the 4000–400 cm^–1^ region with a resolution of 4 cm^–1^ using diffuse reflectance technique, with KBr. High-resolution SEM images were obtained to observe the surface morphology of the films. SEM analysis was conducted using an electron microscope (Jeol, JSM 6060, Japan), according to Goldstein et al.^36^ The samples were placed on stainless steel stubs and gold-coated to examine their morphological characteristics at magnifications of ×5000 and ×60,000.

The determination of the point of zero charge (PZC) is important as it indicates the pH at which the adsorbent surface becomes neutral. The PZC was determined following the procedure outlined by Jorgetto et al.^37^ The methodology involved 11 trials, each with 50 mL of solution, with pH values ranging from 1 to 12. HNO_3_ and NaOH solutions were used to adjust the pH. After adjusting the initial pH, 50 mg of the adsorbent was added to each solution, which were then stirred for 24 h in a shaker (Solab, 222/E, Brazil) at 100 rpm and ambient temperature (25 °C). Afterward, the adsorbents were separated from the solutions, and the pH values were measured to plot the initial pH versus final pH graph. All sample analysis values were compared using Tukey’s test for mean differences, with results considered significant at the 95% confidence level (*p* < 0.05). The analyses were conducted in triplicate.

### 2.6 Effect of pH on the Adsorption Capacity

The pH effect assays on the adsorption capacity of the adsorbents in aqueous solutions were performed using CS/CNTs composite films as adsorbents. For application, adsorbent samples (250 mg) were added to solutions containing a concentration of 200 mg L^–1^ dye, and the pH was adjusted to 2, 4, 6, and 8 by adding 10 mL of 0.1 mol L^–1^ disodium phosphate–citric acid buffer (pH meter Marte, model MB10, Brazil). The solutions were diluted to 100 mL and stirred at 100 rpm using a thermostatic shaker (Fanem 315 SE, Brazil) for 24 h. The liquid was filtered, and the amount of dye remaining in the solution was determined. Adsorption capacities were calculated using eq 2. Adsorption assays were performed in triplicate (*n* = 3)

2 $$q=\frac{\left(C_{0}-C_{f}\right)}{m}V$$

where *C*_0_ and *C*_f_ are the initial and final concentrations of the adsorbate (mg L^–1^), respectively, *m* is the mass of the adsorbent (g), and *V* is the volume of the solution (L).

### 2.7 Kinetics and Equilibrium

Kinetic and equilibrium assays were performed at a more appropriate pH and film composition for each dye. The kinetic studies were performed at 25 °C, with initial dyes concentration of 200 mg L^–1^ at 150 rpm (Nova Ética, 109-1, Brazil). Aliquots were removed at set time intervals (5–60 min). The equilibrium assays were carried out by using a thermostatic agitator (Fanem, 315 SE, Brazil). The temperatures were 25, 35, and 45 °C, and the initial dyes concentrations varied from 50 to 500 mg L^–1^. The adsorption capacity at time *t* (*q*_*t*_) and equilibrium adsorption capacity (*q*_*e*_) were determined by eqs 3 and 4, respectively

3 $$q_{t}=\frac{V\left(C_{0}-C_{t}\right)}{m}$$

4 $$q_{e}=\frac{V\left(C_{0}-C_{e}\right)}{m}$$

where *C*_0_ is the dye initial concentration in the liquid phase (mg L^–1^), *C*_*t*_ is the dye concentration in the liquid phase at time *t* (mg L^–1^), *C*_e_ is the dye equilibrium concentration in the liquid phase (mg L^–1^), *m* is the amount of adsorbent (g), and *V* is the volume of solution (L).

The dye adsorption kinetics behavior was evaluated by pseudo-first-order (PFO), pseudo-second-order (PSO), and Elovich models. The equilibrium isotherms of dye adsorption were fitted using Henry and Freundlich isotherm models. The dye adsorption was also studied by estimation of the thermodynamic parameters, Gibbs free energy change (Δ*G*, kJ mol^–1^), enthalpy change (Δ*H*, kJ mol^–1^), and entropy change (Δ*S*, kJ mol^–1^ K^–1^).^38^ The model parameters were estimated by the fit of the models with experimental data through nonlinear regression by using the Quasi-Newton estimation method. Statistica 7.0 software (Statsoft) was used in the calculations. The fit quality was evaluated by the coefficient of determination (*R* ^2^) and average relative error (ARE).

### 2.8 Desorption and Reuse Assays

Desorption and reuse studies were performed at a more appropriate pH and film composition for each dye. In the reuse experiments, 0.1 g of adsorbent mass was added to 100 mL of dye solution with an initial concentration of 200 mg L^–1^. Desorption experiments were performed in the same volume using NaOH 0.1 and 1.0 mol L^–1^ solutions, respectively. The adsorption and desorption assays were kept under constant shaking for 1 h at 25 °C at each stage. After separation and drying, the films were reused to adsorb the dyes under the same conditions. All of the experimental data were the averages of duplicate determinations.

## 3 Results and Discussion

### 3.1 Characterization of Chitosan

The produced chitosan was characterized in terms of the degree of deacetylation, molar mass, and moisture content. These are key parameters that influence the physicochemical properties of chitosan, such as film or gel formation and mechanical resistance. The chitosan showed a degree of deacetylation of 83.9 ± 1.2%, within the range reported by Moura et al.^39^ (between 70 and 95%). Crini and Badot^15^ highlighted the importance of the degree of deacetylation determination, as it is closely linked to the availability of protonated amino groups for adsorption. The molar mass was 162 ± 3 kDa, also consistent with the range found by Moura et al.^39^ (140–200 kDa). This parameter is related to the mechanical strength of chitosan-based materials. The moisture content was 9.3 ± 0.2% (w w^–1^, wet basis), which was also within the range found by Dotto et al.,^29^ below 10% (w w^–1^, wet basis). The results indicated that chitosan can be used as the precursor material in the development of polymeric films for potential applications in adsorption.

### 3.2 Characteristics of Adsorbent Films

Figure 1 presents the average thicknesses of the CS and CS/CNTs adsorbent films. The results indicate that the thickness of all of the films did not vary significantly (*p* > 0.05). This outcome was considered appropriate, as the same amount of chitosan (2 g, dry weight, dissolved in 50 mL of 1% (v/v) acetic acid solution) was used for all films. Additionally, the amounts of carbon nanotubes added to the film-forming solution were minimal, ranging from 0.1 to 0.5% (w/w). Similar values were reported by Jain et al.^40^ for chitosan–gelatin composite films. The thicknesses of the films found by these authors ranged from 0.072 to 0.103 mm.

**Figure 1:**
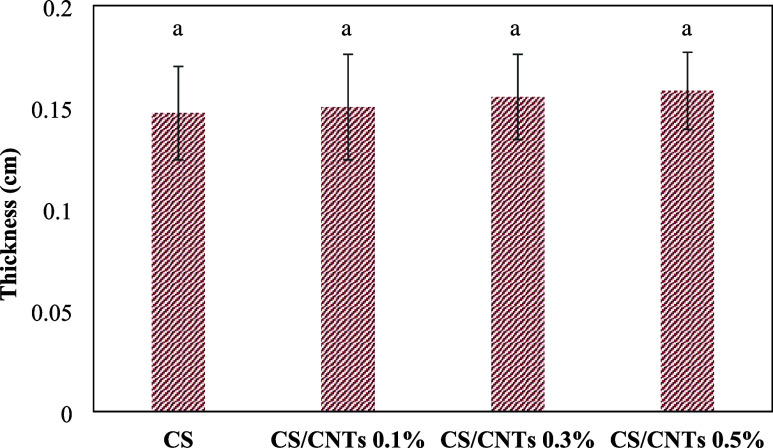
Thickness of the pure chitosan film (CS) and chitosan/carbon nanotube composite films containing 0.1% (CS/CNTs 0.1%), 0.3% (CS/CNTs 0.3%), and 0.5% (CS/CNTs 0.5%) carbon nanotubes.

Figure 2 presents the color parameters of the developed adsorbent films. The control film (CS) exhibited a light-yellow appearance, characterized by a high L* value and a hue angle close to 90°, indicating a yellow tone (Figure 2a). The incorporation of CNTs significantly affected the film coloration (*p* ≤ 0.05), resulting in a progressive darkening as the CNT concentration increased. These changes reflect the reduction in lightness (L*) and shifts in hue angle associated with the increasing presence of carbon nanotubes, which impart a more intense and saturated coloration to the films. Similarly, both a* and b* values decreased, indicating a reduction in the red and yellow chromatic components, respectively (Figure 2b).

**Figure 2:**
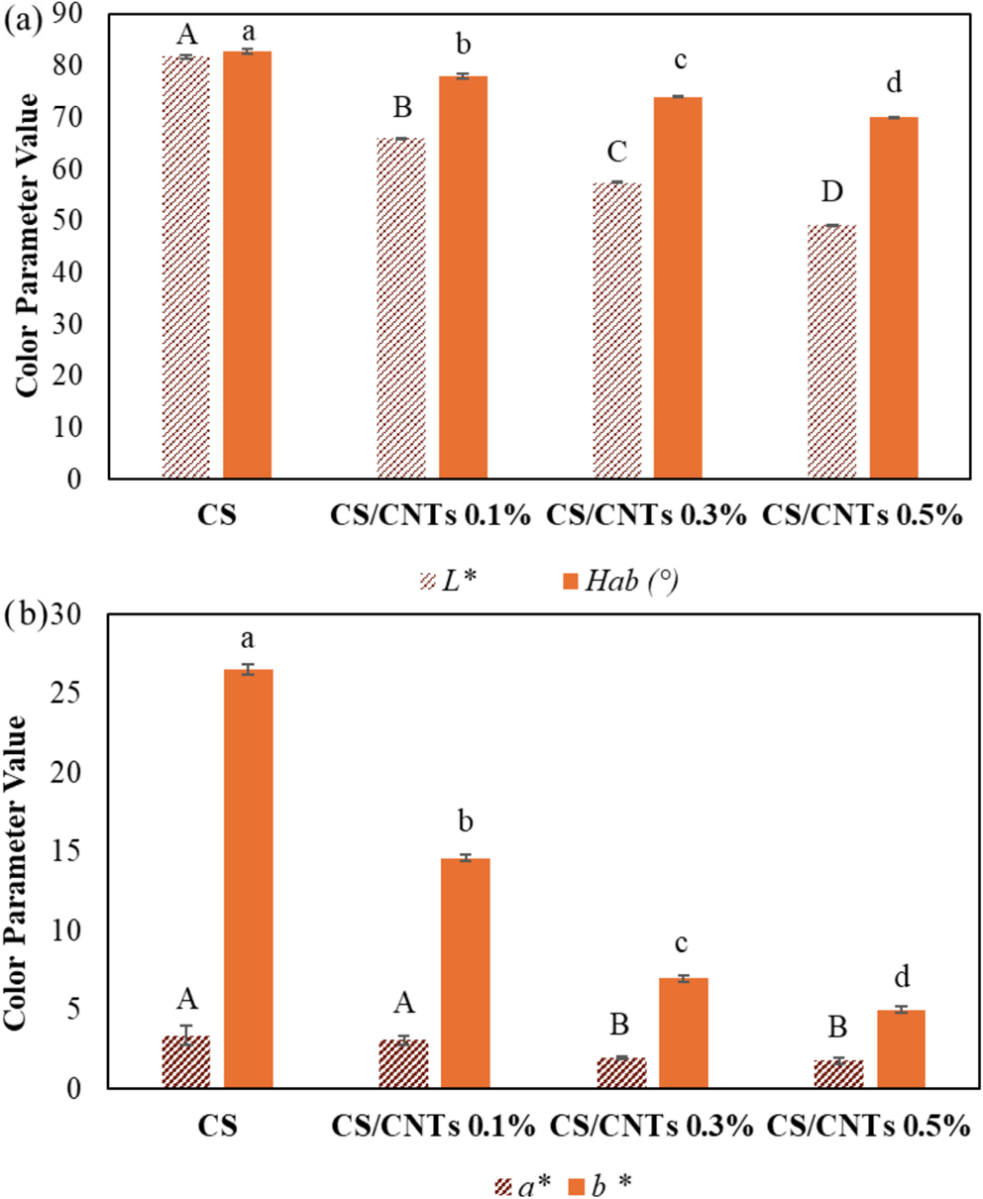
Color parameters: (a) L* and Hue angle; (b) a* and b* of the pure chitosan film (CS) and chitosan/carbon nanotube composite films containing 0.1% (CS/CNTs 0.1%), 0.3% (CS/CNTs 0.3%), and 0.5% (CS/CNTs 0.5%) carbon nanotubes.

The mechanical properties of the films are listed in Table 1. The control film (CS) presented the lowest tensile strength. Similar values were obtained by Moura et al.,^28^ which evaluated the mechanical properties of chitosan films, obtaining tensile strength values ranging from 23.8 ± 0.1 to 39.2 ± 0.1 MPa and elongation values between 9.5 ± 0.1% and 15.7 ± 0.1%. For all of the CS/CNTs composite films (0.1%, 0.3% and 0.5%), the tensile strength values were not significantly different (*p* > 0.05). The addition of CNTs to the polymer matrix led to a significant increase in the tensile strength of the films compared with that of the control CS film. This behavior was similar to that observed for elongation, as all composite films (CS/CNTs) showed an increase in elongation compared to the CS film, with no significant difference observed between the composite films (*p* > 0.05). Rodrigues et al.^41^ also observed an increase in tensile strength for films containing carbon nanotubes, with improvements of 17.6 and 11.3% for films with 0.25 and 0.50 mg of carbon nanotubes, respectively, compared to chitosan single films. However, the elongation break of the nanocomposite films showed a slight decrease relative to the chitosan single film.

**Table 1:** Mechanical Properties of the Adsorbent: Chitosan Single Films (CS) and Films Based on Chitosan/Carbon Nanotubes (CS/CNTs)^a^

films	tensile strength (MPa)	elongation (%)
CS	23.9 ± 2.0^c^	13.9 ± 1.2^c^
CS/CNTs 0.1%	68.0 ± 7.0^a,b^	31.8 ± 1.2^a,b^
CS/CNTs 0.3%	78.2 ± 9.3^a^	29.9 ± 1.4^b^
CS/CNTs 0.5%	83.3 ± 9.8^a^	29.3 ± 1.2^b^

a The mean and standard deviation were calculated (*n* = 3). CS: chitosan single film; CS/CNTs 0.1%: chitosan/carbon nanotubes film with 0.1% nanotubes; CS/CNTs 0.3%: chitosan/carbon nanotubes film with 0.3% nanotubes; CS/CNTs 0.5%: chitosan/carbon nanotubes film with 0.5% nanotubes. Values referenced by different letters in the same column are significantly different (*p* ≤ 0.05).

The results of the X-ray diffraction (XRD) analysis of all films are shown in Figure 3. When observing the film diffractogram, it is noted that all films maintained the profile of semicrystalline material. A similar pattern was observed by Aryaei et al.^42^ According to the authors, the addition of carbon nanotubes into the CS films did not affect the crystalline structure of the films. The chitosan single (CS) film presented irregular intense peaks and an amorphous part, revealing the semicrystalline structure of the polymer. Notably, the chitosan-based films reinforced with CNTs presented more intense peaks at approximately 26 and 45°, which are characteristic peaks of carbon nanotubes and can be attributed to the Bragg indices (002) and (101), which indicate a spacing of approximately 34 nm between the graphene layers. A comparison of the reinforced carbon nanotube films revealed that only the film with the lowest concentration of CNTs presented peaks of different intensities and widths. These differences provide evidence of the organization of the CNT layers. However, no significant changes were observed, indicating that the cylindrical and concentric structures of CNTs were preserved. The incorporation of CNTs increased in the amorphous region of the material, which significantly contributes to contaminant adsorption by enhancing the availability of interaction sites for their attachment.

**Figure 3:**
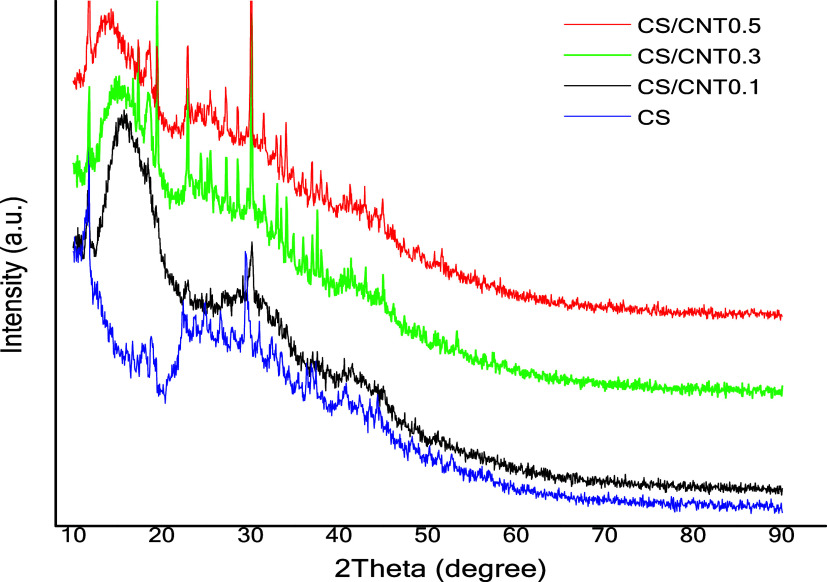
XRD images for chitosan single (CS) film and chitosan with carbon nanotube films (CS/CNT 0.1%, CS/CNT 0.3%, and CS/CNT 0.5%).

Figure 4 shows the TGA thermograms obtained for all of the films. Thermogravimetric analysis was carried out to determine the thermal stability of the developed materials. The percentages of mass loss observed in the thermogram for the chitosan single (CS) film occurred during three thermal events. The first stage occurs between 25 and 115 °C and corresponds to the evaporation of water molecules adsorbed on the material; the second event occurs between 115 and 265 °C and can be related to the decomposition of the amino and hydroxyl groups of the polysaccharide; and the third event between 265 and 450 °C refers to the carbonization of the material organic matter (ash). The CS/CNT films exhibited similar thermal events, although with slightly reduced total mass loss, which is attributed to the lower content of thermally degradable chitosan. These results are consistent with those reported by Rodrigues et al.^41^ that observed the same trend when comparing TGA of chitosan films and chitosan with carbon nanotube films.

**Figure 4:**
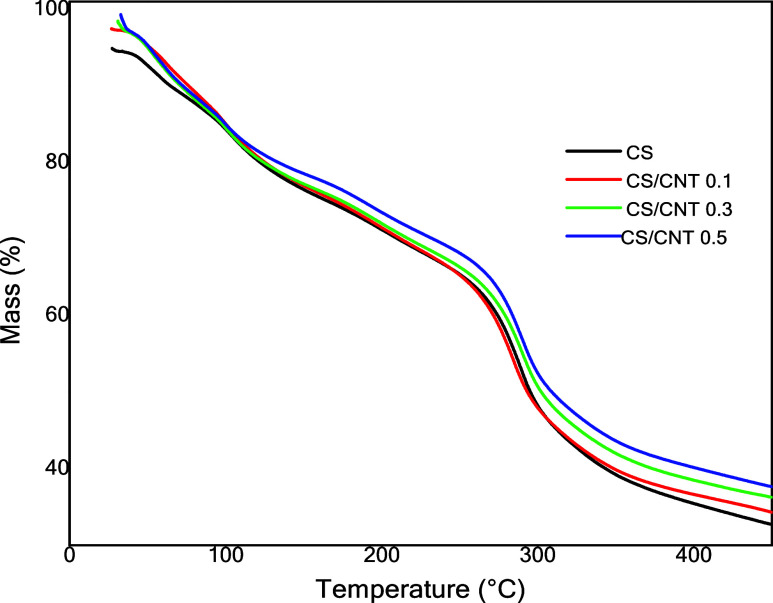
TGA analysis for chitosan single (CS) film and chitosan with carbon nanotube films (CS/CNT 0.1%, CS/CNT 0.3%, and CS/CNT 0.5%).

Figure 5 presents the DSC analysis results for the materials. All films exhibit a pronounced endothermic band in the range of 140–150 °C, attributed to the glass transition (*T*_g_). With the incorporation of CNTs (0.1–0.3%), the endothermic band becomes less intense, and at a concentration of 0.5%, it shifts slightly to lower temperatures and becomes broader. This behavior is attributed to interactions between CNTs and the chitosan matrix, which can interfere with the polymer hydrogen bonding network. Such interactions may disrupt or rearrange the typical inter- and intramolecular interactions of chitosan, leading to modifications in its thermal behavior.^43,44^

**Figure 5:**
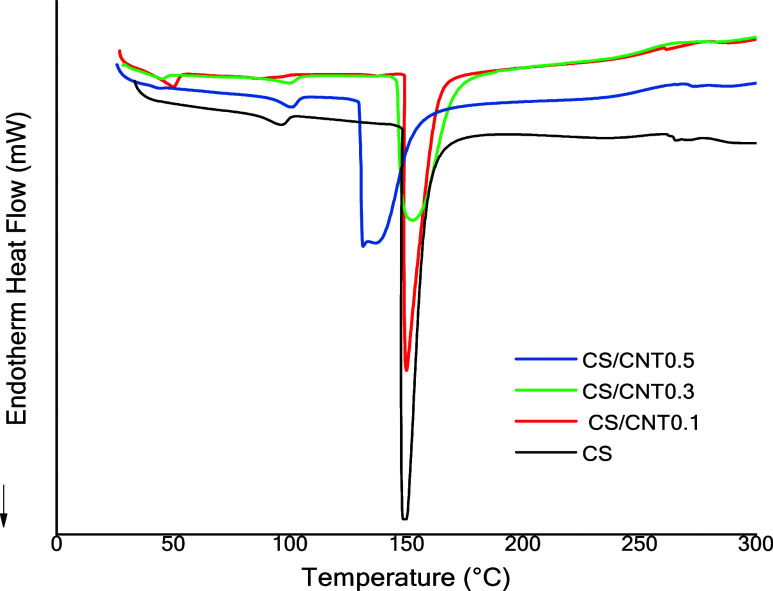
DSC curves for chitosan single (CS) film and chitosan with carbon nanotube films (CS/CNT 0.1%, CS/CNT 0.3%, and CS/CNT 0.5%).

The infrared spectral (FTIR) analysis was carried out to identify the functional groups in the adsorbent precursor materials based on characteristic bands and to analyze the chemical modifications in the composite films. Figure 6 shows the FTIR spectra of the CS films and CS/CNTs composite films. In the spectra, the absorption bands at 1020 and 1450 cm^–1^ appear to be highlighted due to stretching of the C–O bond. These bands are characteristic of chitosan polymer chains.^45^ The FTIR spectra for composite films reinforced with functionalized CNTs (−OH) present bands characteristic of functionalized CNTs, in which an increase in the intensities of vibration bands associated with alcohol is observed at 1140 cm^–1^, and carbonyl bands at 1620 cm^–1^ can be related to carboxylic acids or amides. This increase in intensity as the proportion of CNTs in the material increases can mainly be the result of the induction of dipole moments by creating defects, which are observed in CNTs. Furthermore, bands related to the vibrations of the C=C bond at 1572 cm^–1^, C–H bond at 2930 cm^–1^, and N–H bond at 667 cm^–1^ were observed. The vibration at 2850 cm^–1^ refers to the symmetric stretching of CH_3_, which also indicates that groups were added to the CNTs structure. Rodrigues et al.^41^ reported comparable findings, noting that the vibrational bands in CNTs/chitosan composite films closely resembled those of pure chitosan. The authors attributed this to the low concentration of CNTs (up to 0.5% w/w) and the weak intensity of their spectral signals, which may have been overshadowed by the dominant bands of chitosan, leading to overlap in the spectra.

**Figure 6:**
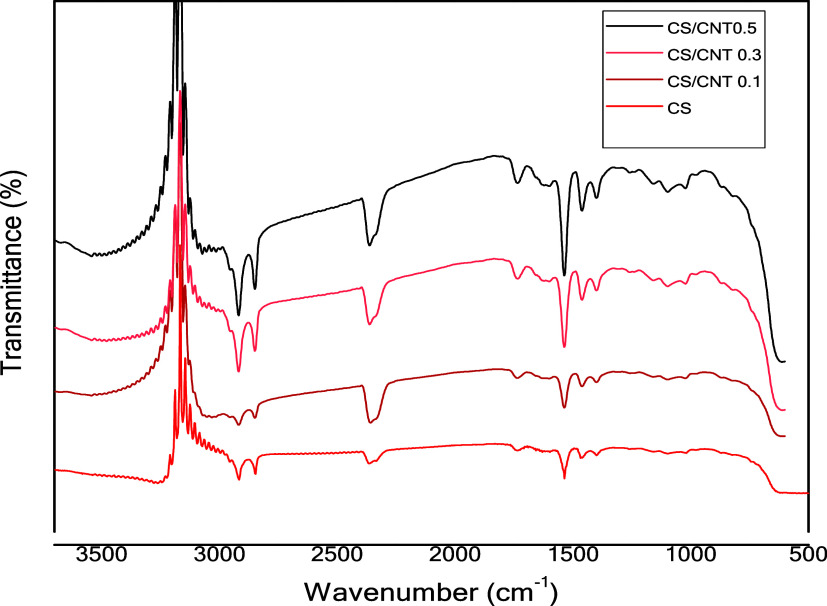
FTIR spectra for chitosan single (CS) film and chitosan with carbon nanotube films (CS/CNT 0.1%, CS/CNT 0.3%, and CS/CNT 0.5%).

Scanning electron microscopy (SEM) images of the films show the surfaces of the adsorbents before (Figure 7a) and after (Figure 7b–d) the addition of nanotubes to the polymer matrix. Morphological images are shown at magnifications of ×5000 and ×60,000 for the CS polymer films (Figure 7a,e), CS/CNTs 0.1% composite films (Figure 7b,f), CS/CNTs 0.3% (Figure 7c,g), and CS/CNTs 0.5% (Figure 7d,h). For the CS film, Figure 7a shows the appearance of aligned lumps; at a higher magnification, Figure 7e shows small grooves on the surface of the lumps. For the composite films reinforced with the 0.1% CS/CNTs nanomaterial (Figure 7b,f), morphological changes that are almost imperceptible to the naked eye are observed, such as the formation of agglomerates in the film structure, indicating that the CNTs are deposited on the surface. For the CS/CNTs 0.3% and CS/CNTs 0.5% films, the alignment of the CNTs and their lengths become more evident and apparent through the formation of clusters in the morphology. All of the films exhibited roughness, which appeared to become more pronounced with increasing CNT content, as qualitatively observed in the SEM images. Shukla et al.^46^ also observed a homogeneous dispersion of CNTs in the chitosan matrix, in contrast to a more homogeneous surface of the chitosan alone matrix.

**Figure 7:**
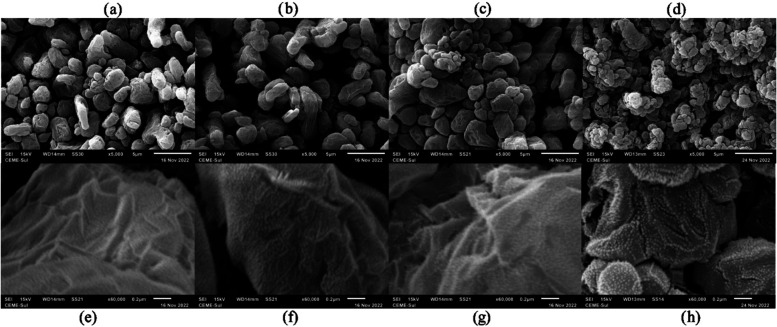
SEM images (magnification ×5000 and ×60,000) of the developed adsorbent: (a, e) chitosan single (CS); and chitosan films containing 0.1, 0.3, and 0.5% carbon nanotubes: (b, f) CS/CNTs 0.1%; (c, g) CS/CNTs 0.3%; and (d, h) CS/CNTs 0.5%.

### 3.3 Adsorption Studies

The point of zero charge (PZC) significantly characterizes how the material adsorbs. The PZC is defined as the pH level that stabilizes after the system achieves equilibrium. As shown in Figure 8, all pH values of the adsorbent films were observed during the study. For the chitosan single films (Figure 8A), the PZC density was 5.8, indicating that the material surface had a positive charge at pH levels below 5.8. In contrast, at pH levels above 5.8, the material was negatively charged.

**Figure 8:**
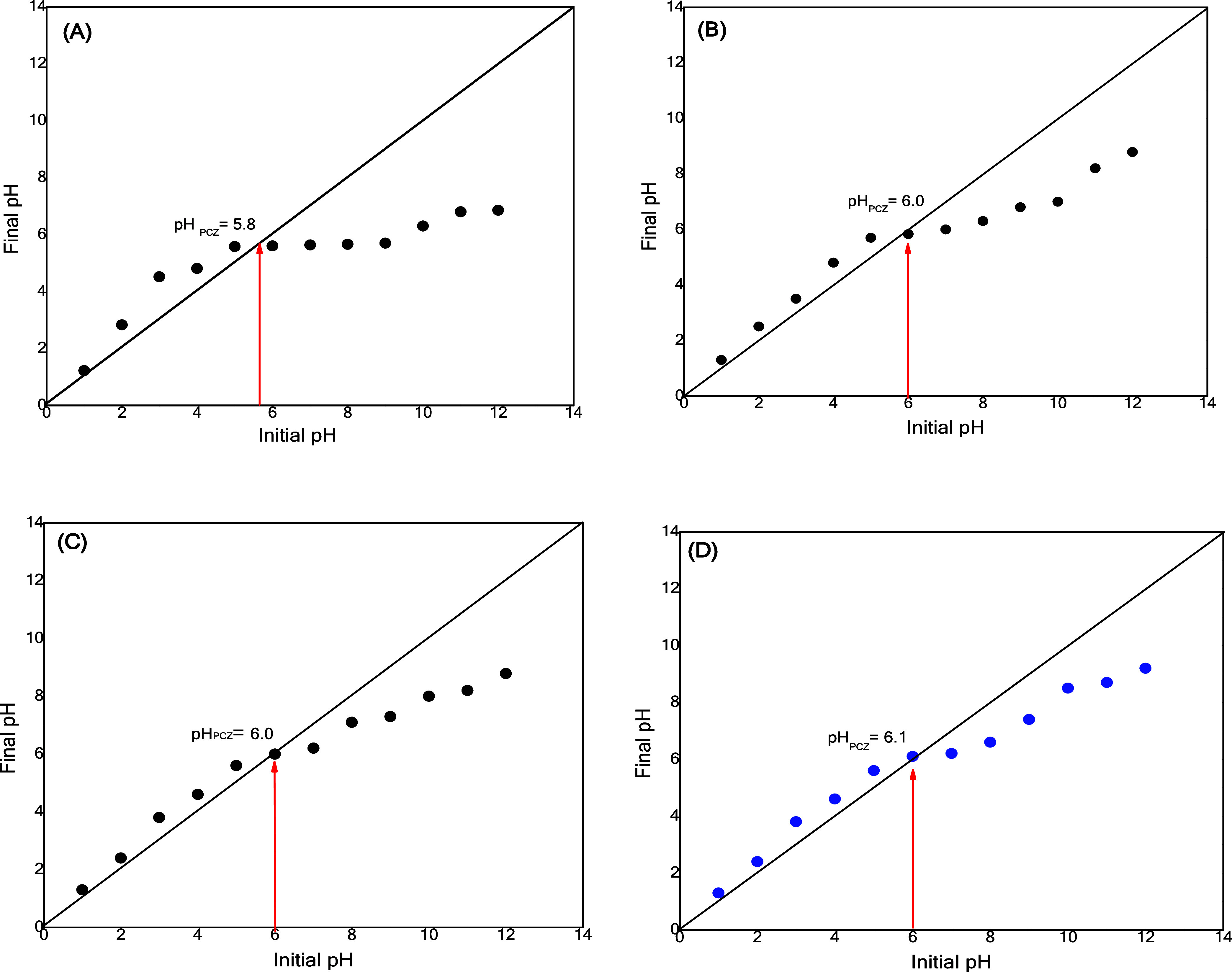
Zero charge points for the adsorbent films: (A) chitosan single film; (B) chitosan film with 0.1% CNTs; (C) chitosan film with 0.3% CNTs; and (D) chitosan film with 0.5% CNTs.

For the other chitosan-based films (Figure 8B–D) containing different proportions of CNT, the PZC pH was close to 6.0, meaning that at pH < 6, the surface will be positively charged, and at pH > 6, the surface will be negatively charged. These results corroborate other similar studies. When a solid adsorbent contacts a liquid solution at a pH lower than the PZC, the surface is positively charged, and many anions are adsorbed to balance the positive charges, increasing the effectiveness of anionic material adsorption.^18^ In aqueous solutions with a pH higher than the PZC, the surface is negatively charged and preferentially adsorbs cations. In this case, the application of adsorbents is recommended for removing cationic materials.^47^ During the dye adsorption process, pH is an important parameter since variations in pH can affect the surface charge of the adsorbent and the ionization degree of the adsorbate. Figure 9 illustrates the effect of the pH on the adsorption capacity (*q*_e_) of the obtained films in cationic and anionic dye solutions. The *q*_e_ values were analyzed from the assays performed at a pH ranging from 2 to 8.

**Figure 9:**
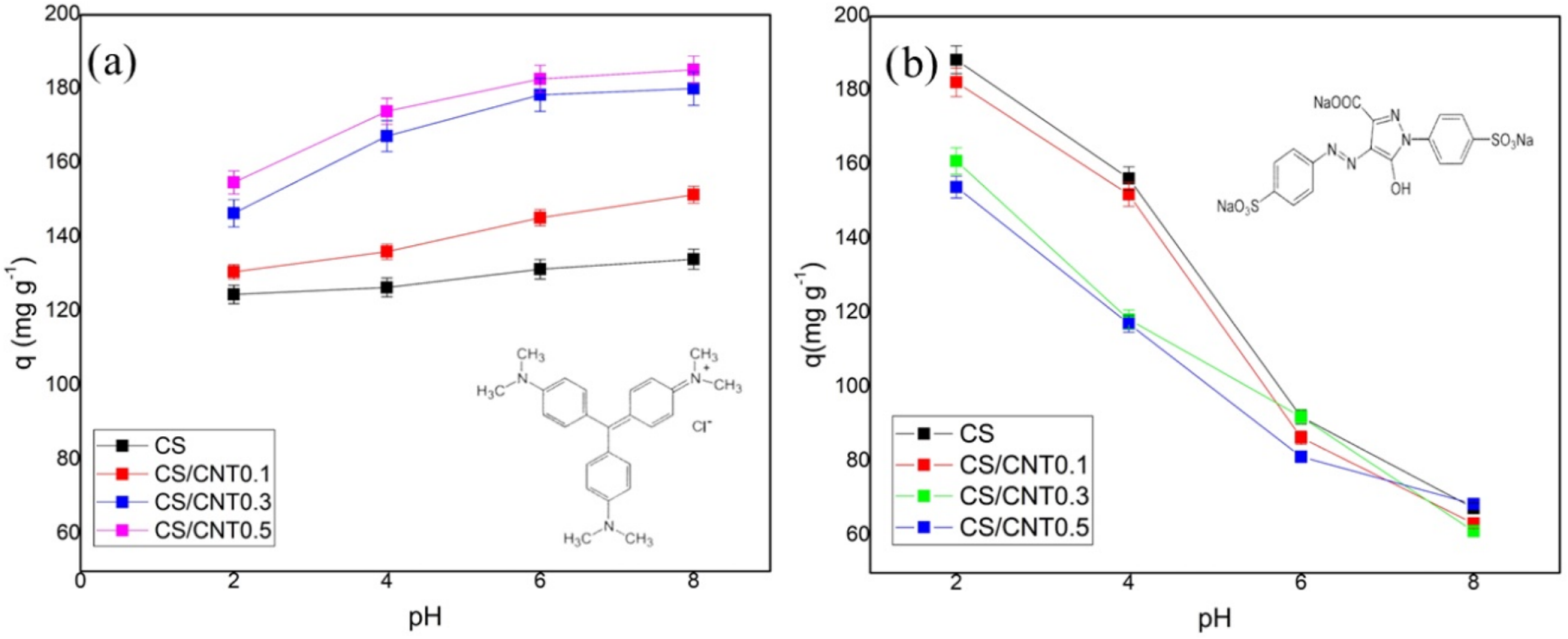
Effect of pH on dye adsorption in chitosan single film (CS) and chitosan/carbon nanotubes films (CS/CNTs) with 0.1, 0.3, and 0.5% carbon nanotubes: (a) crystal violet and (b) tartrazine yellow.

According to the data shown in Figure 9a, the adsorption of crystal violet (cationic dye) in the films is dependent on the pH of the medium. A comparison of the CS film and the CS/CNT films revealed that at all pH values, the composite films presented higher *q*_e_ values than the film without CNTs. This result is attributed to the increased availability of hydroxyl groups on the functionalized CNTs, considering that these functional groups are important sites for adsorption.

The variation in adsorption capacity as a function of the pH of the medium was similar for films containing a higher proportion of CNTs. It was also observed that under acidic conditions (pH < 4), dye adsorption was disfavored, as the functional groups present in CS and CNTs were positively charged, resulting in the repulsion of the crystal violet dye molecules. When the pH increases, the amount of dye adsorbed on the surface of the films increases due to the greater availability of hydroxyl groups. Under basic conditions (pH > 8), no significant changes were observed. Based on the information presented, the adsorption of cationic dyes is favored at pH values above 6.

For the tartrazine yellow anionic dye (Figure 9b), the adsorption capacity of the films was favored by the decrease in pH for all of the films and the reduction in the CS/CNTs ratio. This occurred because, under these conditions, H^+^ ions in the solution tend to protonate the NH_3_^+^ groups of chitosan, facilitating their interaction with the sulfonated groups of tartrazine yellow (negative charge). These results agree with Panda et al.,^48^ which also found that adsorption over chitosan is favored when the pH of the solution is acid for anionic and basic for cationic dyes. In the present study, CNTs were functionalized with (OH^–^) groups, which favored the repulsion of the anionic dye, and the higher the amount of CNTs used in the film, the lower the adsorption of tartrazine dye. Despite the CS/CNT 0.1% film presenting a lower adsorption capacity for yellow tartrazine than the CS film, it was selected for kinetic, thermodynamic, and reusability studies due to its superior mechanical properties.

### 3.4 Kinetic and Thermodynamic Studies

Kinetic and thermodynamic studies were performed using the best film composition and pH condition for each dye: CS/CNT 0.5% at pH 8 for crystal violet and CS/CNT 0.1% at pH 2 for yellow tartrazine. Figure 10 presents the adsorption capacity over time for yellow tartrazine and crystal violet dyes. The results showed that the maximum adsorption capacities were 800 mg g^–1^ for crystal violet and 660 mg g^–1^ for yellow tartrazine. For crystal violet adsorption, the results obtained in this study are higher than those previously reported in the literature. For instance, Olusegun et al.^49^ reported a capacity of 57 mg g^–1^ using niobium oxide coated with a chitosan-activated carbon composite, while Hosseinzadeh^50^ reported a value of 118 mg g^–1^ using a carrageenan/multiwalled carbon nanotube composite. For yellow tartrazine, on the other hand, the values obtained in the preset work are in the range of those reported in the literature. Maximum adsorption capacities of 1065 and 584 mg g^–1^ were reported by Zhang et al.^51^ and Sahnoun et al.,^52^ respectively. These authors also utilized chitosan-based adsorbents to remove yellow tartrazine dye from aqueous solutions.

**Figure 10:**
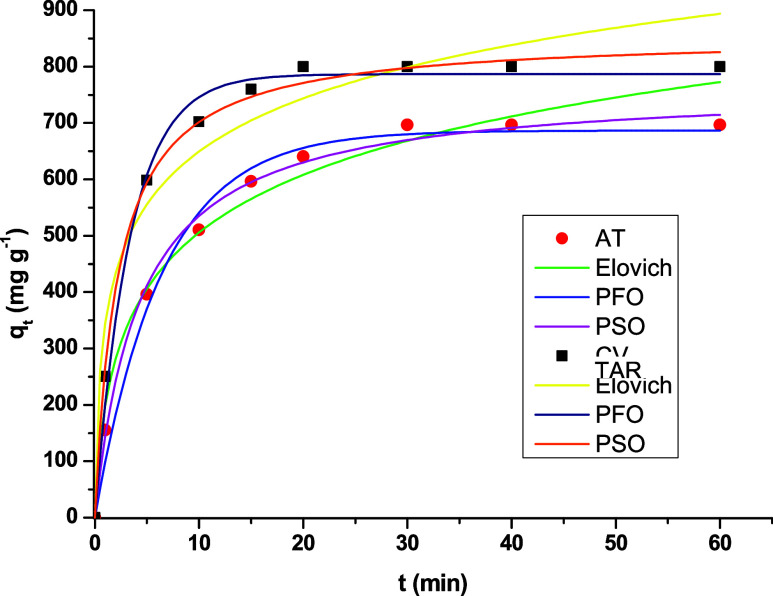
Adsorption kinetics of crystal violet (CV) and yellow tartrazine (TAR) onto chitosan films containing 0.5 and 0.1% carbon nanotubes, respectively.

Experimental data were fitted to the Elovich, PFO, and PSO models to explain the adsorption kinetics. The results of the kinetic analysis are summarized in Table 2, with the corresponding kinetic curves shown in Figure 10. For both dyes, the pseudo-first-order (PFO) and pseudo-second-order (PSO) models demonstrated a good fit to the experimental data, as evidenced by the high *R* ^2^ values and low ARE values. In contrast, the Elovich model yielded the lowest *R* ^2^ value across all of the conditions. Among the models evaluated, the PSO model was considered the most appropriate, as it exhibited the highest *R* ^2^ and lowest ARE values, indicating a superior fit to the experimental equilibrium data under all tested conditions.

**Table 2:** Kinetic Parameters for the Adsorption of Crystal Violet and Yellow Tartrazine Dyes onto Chitosan Films Containing 0.5 and 0.1% Carbon Nanotubes, Respectively

Elovich	*a* (g mg^–1^)	*b* (mg g^–1^ min^–1^)	*R* ^2^	ARE (%)
crystal violet	0.0073	1554.05	0.955	9.23
yellow tartrazine	0.0066	411.46	0.979	6.51
pseudo-first-order	*q*_1_ (mg g^–1^)	*k*_1_ (min^–1^)	*R* ^2^	ARE (%)
crystal violet	786.67	0.296	0.992	4.04
yellow tartrazine	686.66	0.155	0.988	6.75
pseudo-second-order	*q*_2_ (mg g^–1^)	*k*_2_ (g mg^–1^ min^–1^)	*R* ^2^	ARE (%)
crystal violet	856.10	0.00053	0.997	1.98
yellow tartrazine	765.86	0.00030	0.996	2.76

These results are consistent with the findings reported in the literature. Micheletti et al.,^8^ in a review of adsorbents used for the removal of yellow tartrazine from water and wastewater, concluded that the PSO model provided the best fit to adsorption data in the majority of the studies analyzed. Regarding crystal violet, Atmani et al.^53^ and Deepika et al.^54^ also found that the PSO model adequately describes the adsorption of this dye.

Figure 11 presents the equilibrium adsorption capacities for yellow tartrazine and crystal violet dyes at 25, 35, and 45 °C. The results indicate that temperature did not influence the adsorption performance, with maximum capacities remaining around 250 mg·g^–1^ for both dyes. Furthermore, the adsorption curves suggest that the films did not reach saturation within the concentration range tested. The Henry and Freundlich models were applied to identify the most suitable model for describing the equilibrium data and to estimate the corresponding isotherm parameters. Table 3 summarizes the isotherm parameters obtained at each temperature. Both models presented a good fit to experimental data for crystal violet, but the Henry model yielded the highest *R* ^2^ values and the lowest ARE values. Other authors have reported that the Freundlich model better describes crystal violet adsorption using chitosan-based materials.^55^ For yellow tartrazine, the Freundlich model was found to be more appropriate for representing the adsorption behavior. This result is in agreement with the findings of Micheletti et al.,^8^ who identified the Freundlich and Langmuir models as the most commonly used to describe experimental data on yellow tartrazine adsorption.

**Figure 11:**
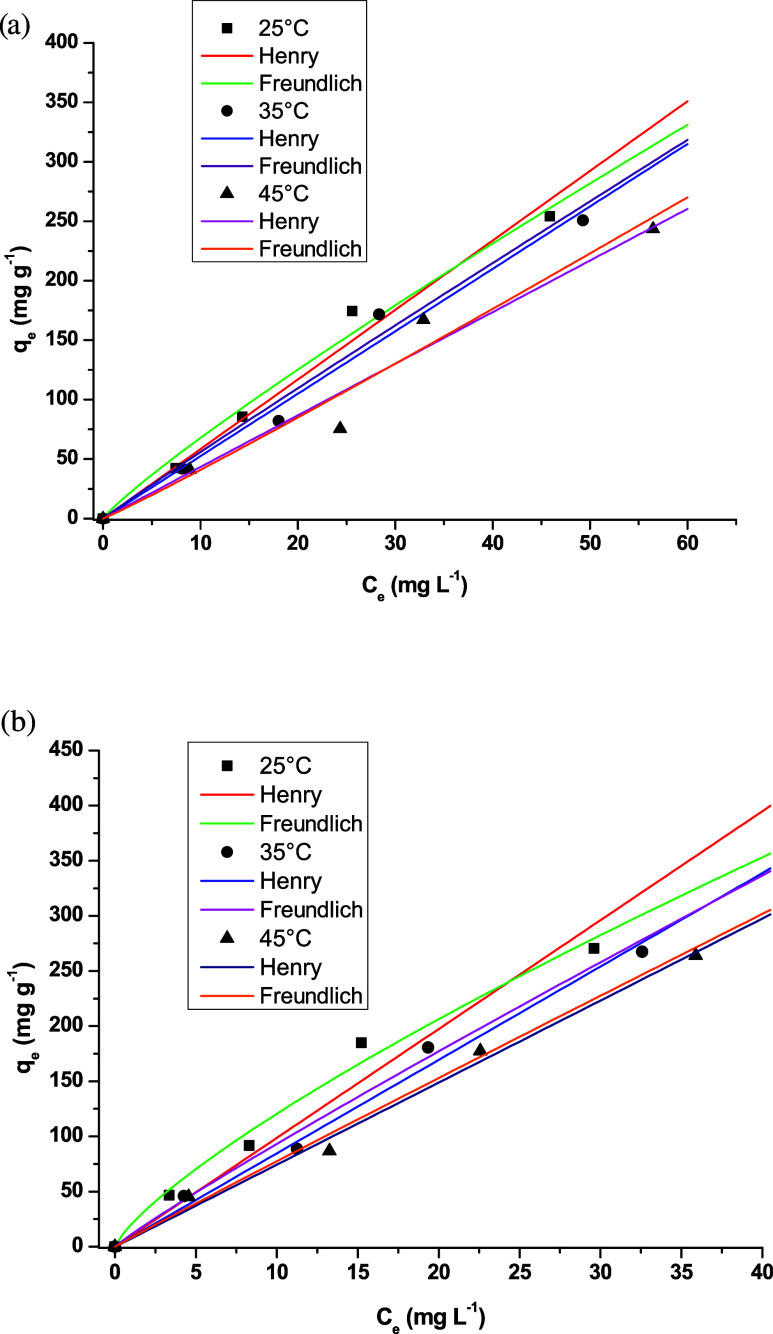
Isotherm models for (a) crystal violet and (b) yellow tartrazine at 25, 35, and 45 °C.

**Table 3:** Isotherm Parameters for Crystal Violet and Yellow Tartrazine Adsorption

	crystal violet			yellow tartrazine		
	temperature					
	25 °C	35 °C	45 °C	25 °C	35 °C	45 °C
Henry						
*k*_H_ (L g^–1^)	5.848	5.25	4.34	9.87	8.47	7.44
*R* ^2^	0.980	0.981	0.961	0.960	0.990	0.991
ARE (%)	4.88	6.72	12.20	13.47	8.19	9.11
Freundlich						
*k*_F_ (mg g^–1^)(mg L^–1^)^−1/*n*^	8.84	5.98	3.63	20.23	11.06	8.11
*n*	1.13	1.03	0.95	1.29	1.08	1.02
*R* ^2^	0.987	0.982	0.962	0.987	0.992	0.991
ARE (%)	8.90	8.14	13.25	7.19	6.37	8.61

Table 4 presents the values of Gibbs free energy change (Δ*G*, kJ mol^–1^), enthalpy change (Δ*H*, kJ mol^–1^), and entropy change (Δ*S*, kJ mol^–1^ K^–1^) for the adsorption process of yellow tartrazine and crystal violet. The positive Δ*G* values indicated that the dyes adsorption by CS/CNTs films was a nonspontaneous process. The values ranging from 21.44 to 22.31 and from 23.65 to 24.35 indicated that the nonspontaneity increased with the increase in temperature. The negative Δ*H* value shows that the adsorption process was exothermic, and the positive Δ*S* value indicated that the disorder in the solid–liquid interface increased during the adsorption process.

**Table 4:** Thermodynamic Parameters for Crystal Violet and Yellow Tartrazine Adsorption

	*T* (°C)	Δ*G* (kJ mol^–1^)	Δ*H* (kJ mol^–1^)	Δ*S* (J mol^–1^ K^–1^)
crystal violet	25	21.44	–8.43	43.67
	35	21.90		
	45	22.31		
yellow tartrazine	25	23.65	–13.17	34.89
	35	23.77		
	45	24.35		

### 3.5 Desorption and Reuse Assays

The desorption and reuse experiments were performed using the best film composition and pH conditions for each dye: CS/CNT 0.5% at pH 8 for crystal violet and CS/CNT 0.1% at pH 2 for yellow tartrazine. The desorption was evaluated using NaOH solutions at concentrations of 0.1 and 1.0 mol·L^–1^. Figure 12 shows the percentage of dye desorption across successive cycles for each system: (a) crystal violet from the CS/CNT 0.5% film and (b) yellow tartrazine from the CS/CNT 0.1% film. The films demonstrated good reusability, as their adsorption capacity decreased by about 12% for crystal violet and 6% for yellow tartrazine after four cycles of adsorption/desorption. Comparable results were presented by Rodrigues et al.^56^ for chitosan-vanadate films. The authors showed that the adsorption capacity of the films was reduced by 20% after 5 cycles using NH_4_OH at 0.01 mol L^–1^ as the eluent.

**Figure 12:**
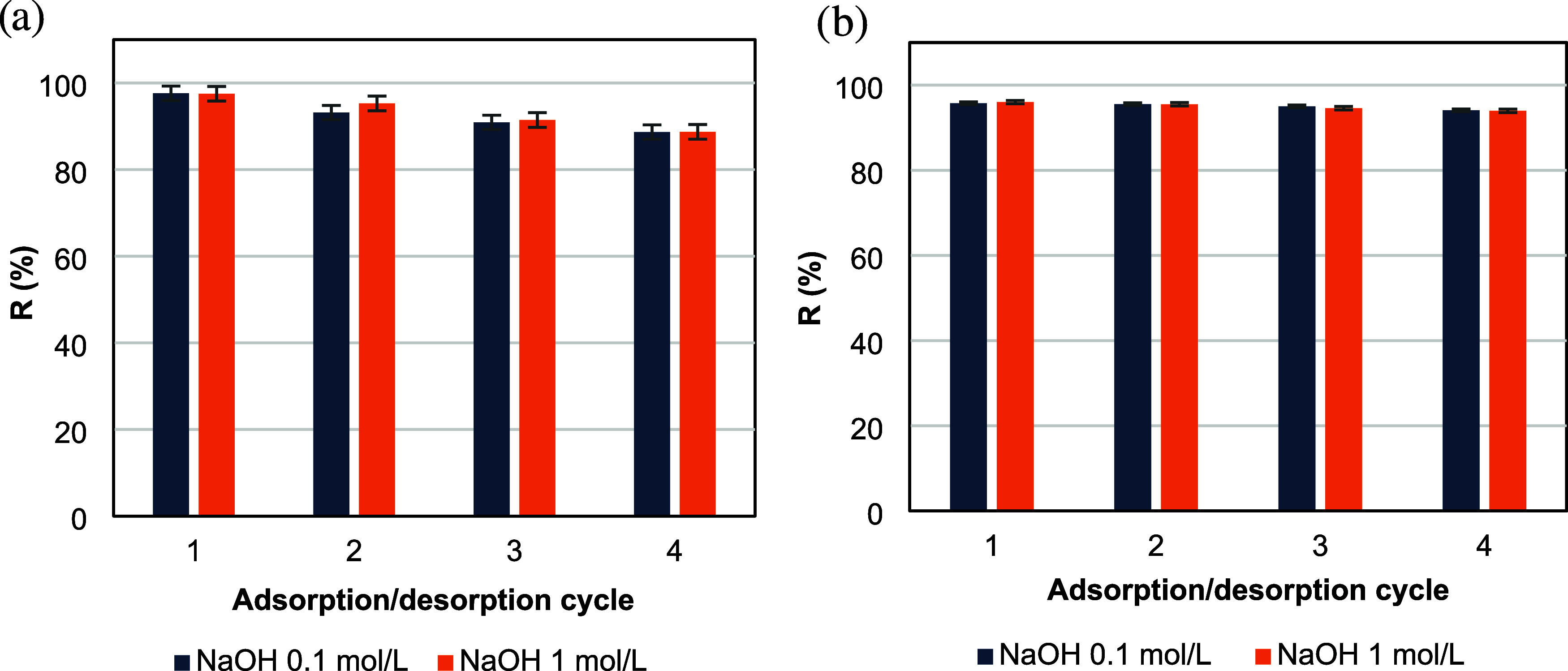
Removal percentage along different adsorption/desorption cycles for (a) crystal violet and (b) yellow tartrazine dyes.

## 4 Conclusions

Chitosan was produced with a degree of deacetylation of approximately 84%, a value within the expected range for the preparation of films with a potential application in adsorption. The films were prepared using a casting methodology in which chitosan (CS) and carbon nanotubes (CNTs) were combined at 0.1%, 0.3%, and 0.5% w w^–1^. The chitosan films with the addition of CNTs exhibited suitable characteristics as adsorbents, including improved mechanical properties. The CS/CNTs 0.5% film exhibited a tensile strength more than three times higher than pure chitosan films (83.3 MPa vs 23.9 MPa) and nearly doubled the elongation at break (13.9 vs 29.3). The incorporation of CNTs resulted in darkening of the films, as indicated by the reduction in L* values and a decrease in the hue angle. TGA analysis showed a reduction in total mass loss due to the lower chitosan content. DSC analysis revealed a decrease in the intensity of the endothermic band and a shift to lower temperatures upon the addition of 0.5% CNTs, suggesting alterations in polymer chain mobility due to interactions with the CNTs. XRD analysis revealed that all films present a semicrystalline structure. The point of zero charge (PZC) was determined to be approximately 5.8 for pure chitosan films and close to 6.0 for CS/CNTs composites, indicating that the surface charge of the materials can be manipulated by the pH to enhance selective dye adsorption. The developed films demonstrated a high efficiency in removing both cationic and anionic dyes from aqueous solutions. For crystal violet (cationic dye), the highest adsorption capacity was achieved at pH 8 using the CS/CNT 0.5% film, reaching 800 mg g^–1^. For yellow tartrazine (anionic dye), the optimal condition was pH 2 with the CS/CNT 0.1% film, achieving a maximum adsorption capacity of 650 mg g^–1^.

Kinetic studies revealed that the pseudo-second-order (PSO) model best described the adsorption processes for both dyes, with high correlation coefficients (*R* ^2^ > 0.99) and low average relative errors (ARE < 5%). Thermodynamic analysis showed that adsorption was exothermic (Δ*H* < 0), with increased entropy (Δ*S* > 0), and nonspontaneous (Δ*G* > 0), with Δ*G* values ranging from 21.44 to 22.31 kJ·mol^–1^ for yellow tartrazine and 23.65 to 24.35 kJ·mol^–1^ for crystal violet, increasing with temperature. Equilibrium data for crystal violet were better described by the Henry model, while yellow tartrazine adsorption followed the Freundlich isotherm. Desorption and reuse tests demonstrated minimal performance loss, with the CS/CNTs 0.5% film retaining 88% of its original adsorption capacity for crystal violet and the CS/CNTs 0.1% film retaining 94% for yellow tartrazine after four cycles. These results confirm the mechanical and chemical stabilities of the materials under repeated use. The films developed in the present work, particularly at 0.5 and 0.1% CNTs loading, were considered reusable and efficient adsorbents for the removal of crystal violet and yellow tartrazine dyes from aqueous solutions, with performance exceeding or comparable to that of similar chitosan-based systems reported in the literature. Future studies could explore the adsorption performance of CS/CNTs films for real industrial effluents, which often contain a variety of contaminants, salts, and interfering substances.

## Abbreviations

CS/CNT: chitosan/carbon nanotubes
CS/CNT 0.1%: chitosan/carbon nanotubes with 0.1% carbon nanotubes in the film-forming solution
CS/CNT 0.3%: chitosan/carbon nanotubes with 0.3% carbon nanotubes in the film-forming solution
CS/CNT 0.5%: chitosan/carbon nanotubes with 0.5% carbon nanotubes in the film-forming solution
